# The Activity of Chosen Antioxidant Enzymes in Ostrich Meat in Relation to the Type of Packaging and Storage Time in Refrigeration

**DOI:** 10.3390/biom11091338

**Published:** 2021-09-10

**Authors:** Olaf K. Horbańczuk, Artur Jóźwik, Jarosław Wyrwisz, Joanna Marchewka, Atanas G. Atanasov, Agnieszka Wierzbicka

**Affiliations:** 1 Department of Technique and Food Product Development, Warsaw University of Life Sciences (WULS-SGGW), 159c Nowoursynowska, 02-776 Warsaw, Poland; j.wywrisz@sggw.edu.pl (J.W.); Agnieszka_Wierzbicka@sggw.edu.pl (A.W.); 2 Institute of Genetics and Animal Biotechnology, Polish Academy of Sciences, 05-552 Jastrzębiec, Poland; aa.jozwik@igbzpan.pl (A.J.); J.marchewka@igbzpan.pl (J.M.); Atanas.Atanasov@dhps.lbg.ac.at (A.G.A.); 3 Ludwig Boltzmann Institute for Digital Health and Patient Safety, Medical University of Vienna, Spitalgasse 23, 1090 Vienna, Austria

**Keywords:** antioxidant enzymes, ostrich meat, type of packaging, storage time

## Abstract

The aim of the study was to investigate the changes in the activity of antioxidant enzymes, i.e., superoxide dismutase (SOD), glutathione peroxidase (GPx) and glutathione reductase (GR), and 2,2-diphenyl-1-picrylhydrazyl (DPPH) free radical scavenging activity in ostrich meat, as influenced by various packaging systems and storage time under refrigeration. Three packaging methods were used: vacuum packaging (VP) and modified atmosphere packaging (MAP) in two combinations of gases, MAP1 (40% O_2_/40% CO_2_/20% N_2_) and MAP2 (60% O_2_/30% CO_2_/10% N_2_). Meat samples were taken from the *M. ilifibularis* (IF) muscles of eight ostriches in each treatment group. The meat samples were stored in a refrigerator in 2 °C and analyzed at days 0, 4, 8, 12 and 16. The lowest level of SOD activity during storage was observed in ostrich muscles packed in vacuum, as compared to MAP1 and MAP2. In turn, the highest increase in GPx activity was recorded in VP, especially up to day 8 of storage, when this parameter reached maximum value (54.37). GR increased up to the eighth day of storage in MAP1 and VP. Between the 12th and 16th days of storage, stabilization of the GR activity level was observed only in VP, while under MAP1, it further decreased. DPPH remained relatively stable until the eighth day of storage and after this period, a decrease in this parameter was recorded, reaching the lowest value on day 12 for all types of packaging systems.

## 1. Introduction

**Figure 1:**
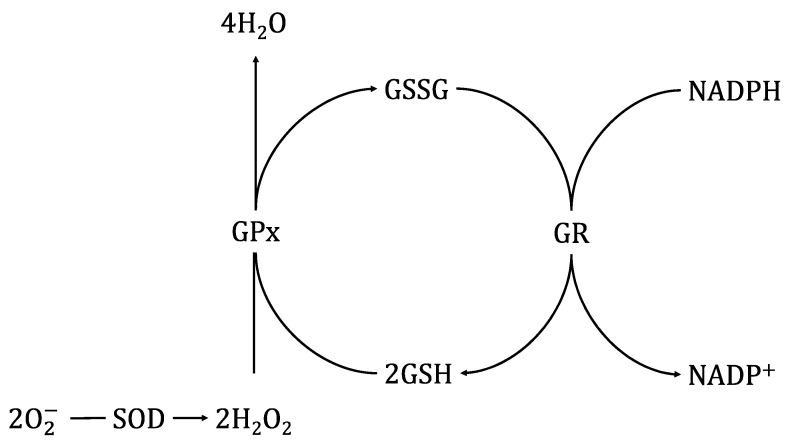
The roles of SOD, GPX and GR in the antioxidant defense enzyme system.

Meat contains multiple initiators and catalysts of oxidation. One of them is heme iron, a major catalyst for the initiation of lipid peroxidation by generation of hydroxyl radicals [1,2,3]. The oxidation level in meat depends also on its content of other prooxidants, especially polyunsaturated fatty acids [4]. It should be stressed that either iron or PUFAs are present in relatively large amounts in ostrich meat [5,6,7,8,9,10,11,12,13]. Thus, this meat type is highly vulnerable to oxidative changes [14,15,16]. Oxidation processes can decrease the nutritional quality of meat and lead to meat quality deterioration, which results in the development of off-flavors and rancidity [17,18]. Some oxidation products can be even toxic for consumers. Therefore, it is very important to prevent the inception of the oxidation processes and establishment of their potentially toxic products (Figure 1). 

Endogenous antioxidant enzymes, e.g., superoxide dismutase (SOD) or glutathione peroxidase (GPx), can control the meat oxidation processes [19,20,21]. For example, GPx can decompose both hydrogen peroxides and lipoperoxides formed during lipid oxidation [22]. This enzyme is also active in post mortem muscle tissue [23] and plays an important role during storage and processing of meat by preventing the oxidation of oxymyoglobin to metmyoglobin [24,25], which leads to negative changes in meat color. Generally, antioxidant enzymes’ activity in meat can differ among production animal species and muscle types, as well as among different packaging conditions and systems [26,27,28]. However, in the currently available literature, there is a shortage of knowledge about the activity of antioxidant enzymes in ostrich meat in relation to the type of packaging and storage time. Thus, the aim of the study was to investigate the changes in the activity of superoxide dismutase (SOD), glutathione peroxidase (GPx), glutathione reductase (GR) and DDPH in ostrich muscles, as influenced by various packaging systems and storage time under refrigeration.

## 2. Material and Methods

### 2.1. Samples and Packaging

Meat samples were obtained from the *Musculus ilifibularis* (IF) of ostriches slaughtered at the age of 10–12 months, weighing between 90 to 95 kg (8 in each group). The IF muscle was excised and the external fat and visible connective tissue were removed from the carcasses 24 h after slaughter. The muscle was cut into 2.5 cm thick steaks starting from the proximal side (sample weight: 150 ± 15 g). Afterwards, the steaks were assigned randomly to one of the three packaging conditions. (a) In vacuum packaging systems, each steak was packaged individually in polyamide/polyethylene (PA/PE) bags (thickness: 90 µm; size: 20/70 mm; CO_2_ permeability: 140 cm^3^/m^2^/24 h; oxygen permeability: 50 cm^3^/m^2^/24 h; water vapor permeability: 6–8 g/m^2^/24 h) 1 min after cutting, and vacuum packed using a Vac-20SL2A packaging machine (Edesa Hostelera S.A., Barcelona, Spain). The in-package vacuum level was 2.5 kPa. (b) For modified atmosphere packaging (MAP) we used two gases combinations, 40% O_2_/40% CO_2_/20% N_2_ (MAP1) and 60% O_2_/30% CO_2_/10% N_2_ (MAP2). The steaks were placed on polyethylene terephthalate/polyethylene (PET/PE) trays (parameters: 187 × 137 × 50 mm), and the film used was a 44 µm thick polyethylene terephthalate/cast polypropylene + antifog (PET/CPP + AF) laminate with maximum oxygen permeability not exceeding 10 cm^3^/m^2^/24 h/bar (EC04, Corenso, Helsinki, Finland). Samples were packed using a M3 packaging machine (Sealpack, Oldenburg, Germany). The packs were stored in a refrigerator at 2 °C for the duration of the experiment, for up to 16 days. Samples collected in three independent replicates were analyzed at 0 (24 h after slaughter), 4, 8, 12 and 16 days of storage.

### 2.2. Superoxide Dismutase (SOD) Assay Procedure

Muscle tissue perfusion was conducted in PBS buffer, at pH 7.4. Homogenization was executed in 5 mL of a 20 mM HEPES buffer (pH 7.2, 1 mM EDTA 210 mM mannitol, 70 mM sucrose per 1 g of tissue), chilled to 4 °C. After that, obtained homogenates were centrifuged at 2500× *g* for 15 min at the temperature of 4 °C. To prevent uncontrolled reaction initiation, it is crucial to store samples on ice until the analysis will be started. Assay procedure was performed with the Superoxide Dismutase Assay Kit, Item No. 706002 (Cayman Chemical Company; Ann Arbor, MI, USA). Measurement of absorbance was made with the help of a microplate reader Synergy4 (Biotek; Winooski, VT 05404 USA). Total activity of SOD was expressed in U/mL.

### 2.3. Determination of Glutathione Peroxidase (GPx)

Perfusion of meat samples was performed using a PBS buffer, at pH 7.4. The tissue was homogenized in 5 mL of buffer, chilled to 4 °C, consisting of 50 mM Tris-HCL, 5 mM EDTA and 1 mM DTT. Centrifuging of samples proceeded at 10.000× *g* for 15 min at the temperature of 4 °C. The supernatant obtained was until the analysis stored on ice. In order to mark the activity of GPx in blood, the material was collected to individual sterile test tubes containing heparin (NH_4_). The samples were centrifuged at 1.000× *g* for 10 min at the temperature of 4 °C. The activity of GPx was marked using the Cayman Chemical Company test. The oxidation reaction of NADPH to NADP^+^ enables the detection of changes in absorbance (λ340). Reading of absorbance and measurement of reaction kinetics was performed using a microplate reader Synergy4 by Biotek. The results were calculated using the Gen5 software. The activity of GPx was expressed in nmol/min/mL. 

### 2.4. Determination of Glutathione Reductase (GR) 

Homogenized were 0.1 g tissues on ice in 0.5–1.0 mL cold assay buffer, or 1 × 10^6^ cells, or 0.2 mL, and in the next step centrifugation was performed at 10,000× *g* for 15 min at 4 °C. The supernatant for assay was collected and stored on ice.

Glutathione reductase was assayed according to the method recommended from the Glutathione reductase (GR) assay kit (Cayman Chemical Company). The assay mixture consisted of 0.1 M potassium phosphate buffer (pH 7.4), 1 mM GSSG, 1 mM EDTA, 0.16 mM NADPH and an appropriate amount of the enzyme source. NADPH oxidation was monitored at 340 nm. The enzyme activity was expressed as nmol/min per mg protein. A standard curve was constructed using pure glutathione reductase (Sigma G4751, St. Louis, MO, USA). Reading of absorbance and measurement of reaction kinetics was performed using a microplate reader Synergy4 by Biotek. The results were calculated using the Gen5 software. The activity of GR was expressed in nmol/min/mL. 

### 2.5. Potential to Scavenge the Free DPPH Radical

The antioxidative activity of the analyzed samples was tested based on an assay procedure using a synthetic DPPH radical (1,1-diphenyl-2-picrylhydrazyl). Muscle tissue perfusion was made with PBS buffer, pH 7.4. Afterwards, 1 g of the muscle was homogenized in 10 mL of cold (4 °C) ultra-pure ethanol. Homogenates were aerated with nitrogen and sealed. The prepared material was for 2 h extracted at the temperature of 40 °C in an ultrasonic bath. The tubes were next cooled and the samples were centrifuged at 4000× *g* for 15 min at 4 °C. To 0.5 mL of thus prepared supernatant, 0.5 mL of an ethanolic solution of 1,1-diphenyl-2-picrylhydrazyl (0.5 mM) was added, that had previously been diluted to ensure its absorbance of ca. 0.9 at the wavelength of λ = 517 nm. The samples were thoroughly mixed and left in a dark, cool place for 30 min for color stabilization. Extinction measurements were conducted using a Cary Win UV spectrophotometer (Varian Inc., New South Wales, Australia), at the wavelength of 517 nm.

### 2.6. Statistical Analysis 

A generalized linear mixed-model analysis (repeated measures ANOVA) was performed on all measured parameters in order to determine the fixed effect of packaging treatment and storage time as a repeated measure, as well as their interaction. Ostriches’ identity (bird number) was included in the model as a random factor. There were no outliers present in the dataset. Normality and homogeneity of residual variance assumptions were checked using the Shapiro test and examination of the normal plot, and these were met by all variables under investigation. PROC GLIMMIX of SAS v 9.4 (SAS Institute Inc., Cary, NC, USA) including Tukey’s adjustment option was used to conduct the analysis. The validity of the models was tested using Akaike’s information criterion. The least square means for all significant effects in the models (*p* ≤ 0.05) were computed using the LSMEANS option. For all analyses, results are reported as means ± standard error of the mean (SEM). 

## 3. Results and Discussion 

**Figure 2:**
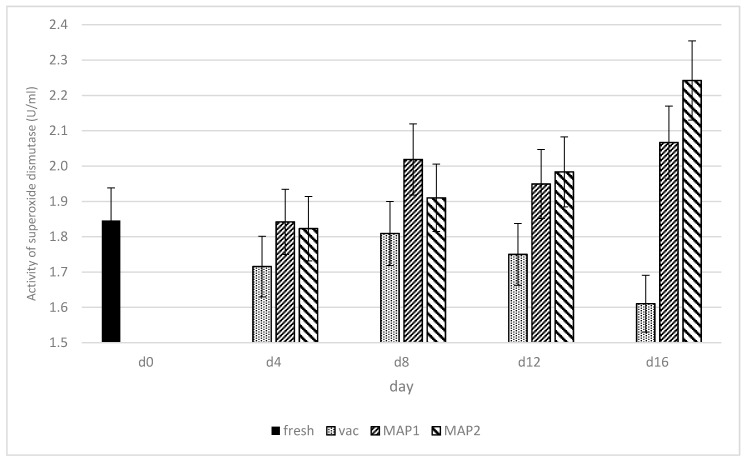
Changes in the activity (U/mL) of superoxide dismutase (SOD) in ostrich meat as influenced by the type of packaging and storage time in refrigeration.

**Figure 3:**
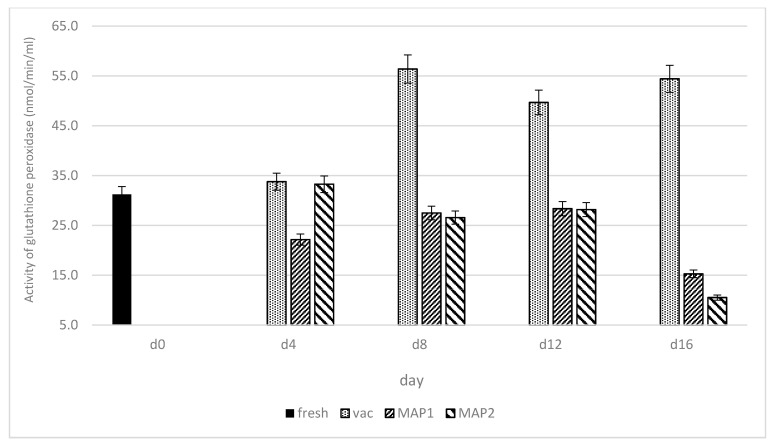
Changes in the activity (nmol/min/mL) of glutathione peroxidase (GPx) in ostrich muscles as influenced by the type of packaging and storage time in refrigeration.

**Figure 4:**
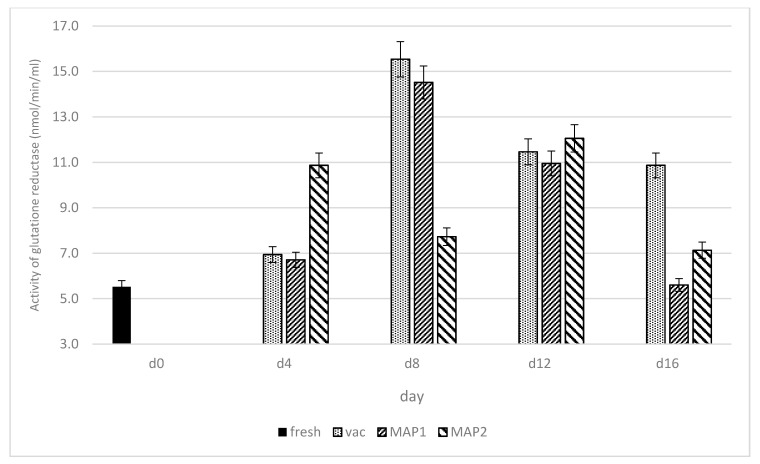
Changes in the activity (nmol/min/mL) of glutathione reductase (GR) in ostrich muscles as influenced by the type of packaging and storage time in refrigeration.

**Figure 5:**
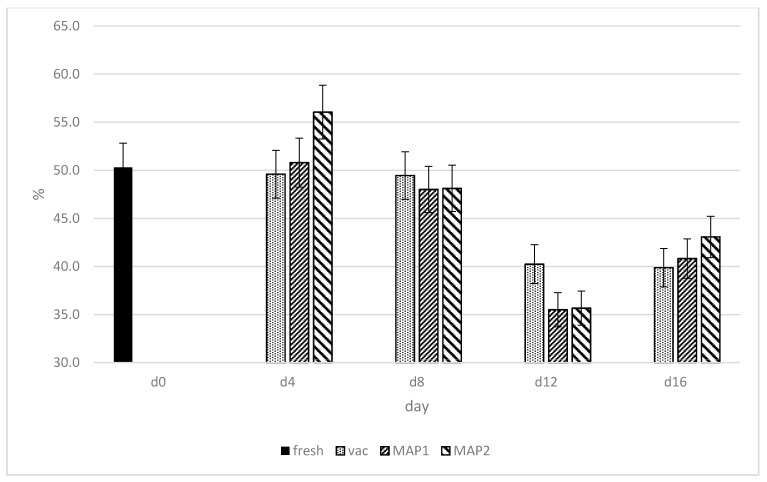
Changes (%) in the activity of DPPH free radical scavenging in ostrich muscles as influenced by the type of packaging and storage time in refrigeration.

The changes in the activity of superoxide dismutase (SOD), glutathione peroxidase (GPx), glutathione reductase (GR) and potential of free radical scavenging (DPPH), as influenced by various packaging systems and storage time under refrigeration are presented in Figure 2, Figure 3, Figure 4 and Figure 5. 

Superoxide dismutase.

Meat contains endogenous antioxidants, as the live cells have several mechanisms of protection against oxidative processes, including antioxidant enzymes such as superoxide dismutase (SOD), which plays an important role in protecting against damage by the superoxide anion radical [29]. According to Filgueras et al. [30], the extent of oxidative processes in meat is dependent on the balance between concentration of lipidic substances sensible to peroxidation (i.e., polyunsaturated fatty acids or PUFA) and antioxidant enzymes such as SOD or glutathione peroxidase (GPx). The activity levels of superoxide dismutase (SOD) are presented in Figure 2. Overall, the lowest level of SOD activity during the entire storage time was observed in ostrich muscles packed in vacuum, as compared to MAP1 and MAP2 packing methods. The lowest value of SOD was observed in vacuum on the 16th day of storage (1.65 U/mL), as compared to MAP1 (2.05 U/mL) and MAP2 (2.24 U/mL). Under MAP1 and MAP 2 packaging, SOD levels increased over the storage days in the current experiment. Under MAP1 SOD activity was significantly lower as compared to days 8 (2.00 U/mL),12 (1.95 U/mL) and 16 (2.05 U/mL) of the experiment. Under MAP2, SOD activity was significantly lower on days 0–12 (1.85–1.99 U/mL), as compared to day 16 (2.24 U/mL) of the storage. On the contrary, the level of SOD in vacuum packaging decreased significantly between day 0 (1.85 U/mL) and day 16 (1.65 U/mL). Similar SOD activity was shown in studies of rhea meat by [31], ranging from 1.77–2.04 U/mL. In early studies by Renerre et al. [23], higher SOD activity in bovine muscle (3.0 units 1 day postmortem) was found. It is probable that in vacuum packaging method, due to anaerobic conditions, the generation processes of free radical formation were reduced, whereas in MAP1 and MAP2, a rise in this parameter during storage was observed (Figure 2). It may indicate an increased, more intensive dismutation process and converting free radicals into a hydroxyl radical. Similar tendencies of decreased activity of SOD in VP during storage was shown by Pastsart et al. [31] in beef muscles at day 10 as compared to day 0. Moreover, Adeyemi [32] recorded decreased SOD activity for the vacuum-packed goat meat stored up to 8 days. However, results of our study are different from those obtained for vacuum packed beef from Belgian Blue cattle stored at −1 °C for up to 28 days after 7 days of display [33].

### 3.1. Glutathione Peroxidase (GPx) and Glutathione Reductase (GR)

Glutathione is the major metabolite involved in determining cellular redox state, while the enzymes responsible for glutathione metabolism are glutathione peroxidase (GPx) and glutathione reductase (GR) [34,35,36]. An antioxidant function has been classically assigned to these enzymes. The activity levels of GPx and GR are presented in Figure 3 and Figure 4. The highest increase in GPx activity occurred in ostrich muscles packed in vacuum, from day 4 (33.89 nmol/min/mL) of storage to day 8, when this parameter reached maximum value (54.37 nmol/min/mL). After day 8 of storage, the GPx activity stabilized in VP until the end of storage time (day 16; 48.4 nmol/min/mL) at a relatively high level in comparison to ostrich muscle on day 0 (31.67 nmol/min/mL). These data provide evidence that GPx may protect muscle tissue against degradation, and in consequence, against the deterioration of the meat quality. In research conducted by Watanabe et al. [37], the increasing activity levels of GPx during storage time were consistent in fish stored up to 5 days at 4 °C. According to these authors, a decrease in GPx activity can be related to the hydrolysis of this enzyme by intracellular proteinases during storage or due to enzyme denaturation [37]. Similar tendencies were shown by Daun [38], where GPx remained stable after storage time in vacuum packed beef (1.9 U/g) stored in −20 °C up to 14 days, and vacuum-packed pork up to 4 days stored in 4 °C. The increase in the activity of GPx enzymes in VAC may be caused by a higher affinity for the generation of secondary free radicals [39]. The rise in GPx may also be associated with greater protection against the occurrence of oxidation processes, which is not observed in MAP1 and MAP2. Overall, the GPx activity in MAP1 and MAP2 was lower and more stable, as compared to vacuum packaging, especially until day 12, while between 12 and 16 days of storage, a significant decrease in its activity was observed (Figure 3). On day 16, meat samples packed in MAP showed the lowest activities for GPx, which was an opposite trend to the superoxide dismutase activity (Figure 2). The observed tendency for the decrease in GPx activity in both MAPs was similar to the decrease found in chicken meat during refrigerated storage by Gheisari [40]. In our study, the higher activity of GPx for meat packed in VAC may also indicate that under vacuum conditions, antioxidant protection in the meat maturation processes is taken over by glutathione reductase (GR). In case of GR, its increased activity in ostrich muscles up to the eighth day of storage in vacuum (15.51 nmol/min/mL) and MAP1 (14.90 nmol/min/mL) was observed, which was probably associated with the existence of the muscle tissue protection mechanism against degradation and redox process reverse occurring in the ostrich meat during storage (Figure 4). GR activity under the storage continued after day 8 to day 12 indicated a decrease level of GR in MAP1 and VP (10.72 nmol/min/mL and 10.35 nmol/min/mL, respectively). However, the stabilization of the GR activity level between the 12th and 16th days of storage was observed only in vacuum, while under MAP1, further decrease in GR (5.66 nmol/min/mL) occurred (Figure 4). 

### 3.2. DPPH

The percentage activity of DPPH free radical scavenging is presented in Figure 5. The potential of DPPH in ostrich meat was maintained on a relatively stable level up to the eighth day of storage, with no significant differences observed for MAP1 and vacuum packaging systems. These data suggest that during storage, free radicals were neutralized by increasing the activity of the antioxidant investigated enzymes. After this period (8 day), a decrease in DPPH was observed and the lowest level of this parameter was recorded on day 12 either in vacuum or MAP1 and MAP2 (Figure 5). The results of DPPH activity levels in the current study were generally consistent with those of Fasseas at al. [41], where overall DPPH activity of investigated beef meat samples decreased within storage time from 1 to day 12.

## 4. Conclusions

In conclusion, the current study was designed to assess the changes in the activity of the antioxidant enzymes superoxide dismutase, glutathione peroxidase and glutathione reductase, as well as DPPH, in ostrich muscles in relation to various packaging systems and storage time under refrigeration. Based on SOD, GPx and GR activity levels, the antioxidant protection potential of the cells in ostrich muscles generally decreased after 12 days of storage in all packaging systems except for MAP1 and MAP2 in the case of SOD and GPx in VP. The increase in the activity of GPx enzymes in VP may be caused by a higher affinity for the generation of secondary free radicals. In turn, the DPPH in ostrich meat was maintained on a relatively stable level until the eighth day of storage and after this period, a decrease in this parameter was reported, achieving its lowest value on day 12 under all types of packaging systems. 

## Data Availability

Not applicable.
